# First-generation students’ underperformance at university: the impact of the function of selection

**DOI:** 10.3389/fpsyg.2015.00710

**Published:** 2015-05-28

**Authors:** Mickaël Jury, Annique Smeding, Céline Darnon

**Affiliations:** ^1^Laboratoire de Psychologie Sociale et Cognitive, CNRS, UMR 6024, Université Clermont Auvergne, Clermont-Ferrand, France; ^2^Laboratoire Interuniversitaire de Psychologie-Personnalité, Cognition et Changement Social, Université Savoie Mont Blanc, Chambéry, France; ^3^Institut Universitaire de France, Paris, France

**Keywords:** university, social class, achievement gap, threat, vigilance, eye-tracking

## Abstract

According to recent research, university not only has the role to educate and train students, it also has the role to select the best students. We argue that this function of selection disadvantages first-generation students, in comparison with continuing-generation students. Thus, the mere activation of the function of selection should be sufficient to produce achievement differences between first-generation and continuing-generation students in a novel academic task. Furthermore, we propose that when the function of selection is salient, first-generation students would be more vigilant to a cue that may confirm their inferiority, which should explain their underperformance. In the present experiment, participants were asked to complete an arithmetic modular task under two conditions, which either made the function of selection salient or reduced its importance. Participants’ vigilance to a threatening cue (i.e., their performance relative to others) was measured through an eye-tracking technique. The results confirmed that first-generation students performed more poorly compared to continuing-generation students only when the function of selection was salient while no differences appeared in the no-selection condition. Regarding vigilance, the results did not confirm our hypothesis; thus, mediation path could not be tested. However, results indicated that at a high level of initial performance, first-generation students looked more often at the threatening cue. In others words, these students seemed more concerned about whether they were performing more poorly than others compared to their continuing-generation counterparts. Some methodological issues are discussed, notably regarding the measure of vigilance.

## Introduction

The university is an institution defined as a system that gives the same chances of success to every student, regardless of his or her social background. However, a lot of studies show that low social-class students have poorer chances to succeed in the educational system, including university, in comparison with their high social-class counterparts (White, 1982; Sirin, 2005; OECD, 2014). How can such inequalities persist despite efforts to make university a place where everyone should have the same chances to succeed? In the present paper, we examine how the function of selection fulfilled by the university system can at least partially contribute to reproducing unequal chances of success for students from low and high social-class backgrounds.

Recent research has documented that, in Western societies, university fulfills two distinct functions (Dornbusch et al., 1996; Darnon et al., 2009, 2012; Smeding et al., 2013). Indeed, the functional perspective of education presented by Dornbusch et al. (1996) argues that the educational system first has to “teach the cognitive skills necessary to perform occupations” (p. 405). In other words, university has a function of education with the official role to teach and develop students’ skills and knowledge. Besides this function, the educational system should also “attempt to provide a rational means of selecting persons in order that the most able and motivated persons are sorted into the highest status positions” (p. 405). Thus, university also has to identify the best students and reward them with degrees, a less explicit and less official function called the function of selection. To fulfill this function, policymakers proposed selecting students on the strict basis of their merit to ultimately assign students to the “place where they belong” (Bourdieu et al., 1990; Darnon et al., 2009). The role of university is thus to identify the more deserving/talented students who should eventually graduate, possibly with honors, and who should have access to high-status jobs.

To “properly” identify the best students among others, university can use different kinds of selection instruments and procedures. In some universities, students have to run in-curriculum competitive exams with *numerus clausus* (for consequences on students’ motivation, see Sommet et al., 2013). In some others, the selection step is at the admission level (e.g., in Harvard, only 5.9% of the applicants were selected to enter in the curriculum in 2014). Thus, although the selection process does not take the same form in all systems, it is highly present in most universities.

Despite the institutional discourse calling for equality in opportunities, it seems that the function of selection consistently acts in favor of high social-class students and at the disadvantage of low social-class students, hence contributing to the social reproduction of inequalities (Bourdieu and Passeron, 1964). Indeed, high social-class students represent a higher percentage of university graduates and/or get higher grades than low social-class students (OECD, 2014). They are also over represented in highly selective colleges (Carnevale and Rose, 2003; Alon, 2009; Hearn and Rosinger, 2014) as well as in graduate school (Kniffin, 2007). These discrepancies lead some authors to argue that the promise of meritocracy—underlying the university selection—is “unfulfillable” (Mijs, 2015).

Several examples in the literature illustrate that how universities intrinsically function and operate has a distinct effect on students’ experiences depending on their social-class. Indeed, if such a system provides a rather comfortable environment for high social-class students, it also shapes low social-class students experiences in a way that restrains their success. Indeed, the university system promotes values, ideas, and language use that are more widely shared by dominant group members (e.g., high social-class students) than by dominated group members (e.g., low social-class students). For example, Bourdieu and Passeron (1964, 1970) and Bourdieu et al. (1990) originally assumed that students from low social-class background have fewer chances to succeed at university due to their lower economic capital (i.e., financial resources) and their lower cultural capital (i.e., cultural characteristics valued in the system, which are conveyed through speech, attitudes, knowledge, and behaviors). Low social-class students might be less likely to succeed compared to high social-class students because their parents have not taught them implicit rules and norms that could help them know how to behave and succeed in the university system (for empirical evidence, see Gaddis, 2013; Calarco, 2014).

In line with this idea, the recent work of Stephens et al. (2012a,b,c, 2014) has significantly contributed to documenting how intrinsic characteristics of university functioning can exert an influence on low social-class students’ higher education experience. In their work, the authors examined how the university system contributes to the performance gap between first-generation students (i.e., students whose parents do not have a college degree) and continuing-generation students (i.e., students whose one or both parents have a college degree). Authors mainly argue that the independent values promoted in the university context (e.g., autonomy, development of one’s own way of thinking) should be in conflict with those of first-generation students and that this discrepancy should explain first-generation students’ underachievement. Indeed, due to their working-class socialization—contexts in which interdependent values (e.g., learning from others, working together) are usually promoted—first-generation students develop an interdependent self-concept that mismatches with the values promoted within the system. In a series of studies, the authors provided empirical evidences that this mismatch leads first-generation students to perform more poorly and to have a poorer emotional experience at university in comparison with continuing-generation students. When this mismatch is not experienced (when interdependent values are promoted) these differences in performance and emotional experience disappeared (Stephens et al., 2012a,c).

In a different field of research, the stereotype threat literature also offers important illustrations of the difficulties encountered by low social-class students at university. According to this literature, when negative stereotypes are activated, stigmatized individuals can experience “stereotype threat”—a phenomenon that results in an aversive experience and in reduced performance (Steele and Aronson, 1995). In particular, when attending university, low social-class students are targeted by a negative stereotype (Berjot and Drozda-Senkowska, 2007), which can be threatening and impairs their psychological functioning (Schmader et al., 2008). Croizet and Claire (1998) provided initial empirical evidence that this phenomenon can affect low social-class students in the university context by showing that these students performed more poorly than their high social-class counterparts only when the task was presented as diagnostic of their intellectual ability. Additional studies consolidated their results by showing that (i) low social-class students seem to face this threat particularly when their social class was salient (Spencer and Castano, 2007) and (ii) they experienced more anxiety and a lower level of academic identification when the task was presented as being diagnostic of intellectual ability rather than when it was not (Harrison et al., 2006).

Taken together, these different mechanisms (i.e., social reproduction, cultural mismatch, stereotype threat) illustrate how the university functioning restrains the chances of success of low social class students and favors those of high social class students. They also share some common characteristics and processes. Indeed, in the typical situations examined in this research, the university context questioned low social-class students’ legitimacy and sense of belonging. We argue that the function of selection is precisely the reason why, in this system, “dominant” norms and high social-class values are promoted while negative stereotypic expectancies targeting low social-class students are regularly activated. Consequently, the university system might induce a threat for low social-class students’ social identity—a threat that is particularly likely to occur when people are led to think that they might be eliminated from the system. Indeed, the structural “need to select the best students,” the function of selection, by being at the disadvantage of low social-class students, should favor this general threat-inducing context and, consequently, drive low social-class students’ negative experiences at university.

As a preliminary step to support the hypothesized role of the function of selection on the social-class achievement gap, Smeding et al. (2013) manipulated the selective function of an exam and tested its effect on the performance of low and high social-class students. The exam was either presented as a tool for education and mastery (e.g., “this exam has been designed to help students in their long-term learning”) or as a tool for selection (e.g., “this exam has been designed to compare students in their long-term learning”; see Smeding et al., 2013). Low social-class students performed more poorly compared to high social-class students on the selection-oriented exam, a difference that did not appear when the exam was presented as a tool to train and educate students (i.e., mastery-oriented exam). These results support the idea that the function of selection, when salient in exam situations, might contribute to the social-class achievement gap. As such, this line of research can be understood as a structural (Dornbusch et al., 1996) perspective on intergroup differences in academic performance, a different yet very complementary level of analysis to those generally adopted in the stereotype threat literature. Indeed, stereotype threat research showed that the activation of a negative stereotype targeting their social group is the process that makes stigmatized individuals experiencing a disruption in their psychological functioning. The line of research presented here shows that the mechanism responsible of that disruption has to be found in the structural functioning of the institution (i.e., its function of selection) and that the psychological experiences of stigmatized students (i.e., low social-class students) can be impaired without directly activating the negative stereotype of their group.

The present research aims to extend Smeding et al.’s (2013) work in an important way. In this work, the function of selection was activated via the function attributed to the exam situation. Exam situations are very intense experiences in students’ lives (Crooks, 1988), as they are used as a criterion to decide whether students can obtain a degree. This is probably why Bourdieu and Passeron (1964, 1970; see also Delandshere, 2001; Leathwood, 2005) considered them as particularly involved in the social reproduction phenomenon. Moreover, exams are supposed to diagnose students’ ability to succeed and are particularly susceptible to create a threatening environment in which the difference between groups is likely to be observed (see Danaher and Crandall, 2008). Therefore, one can think that the effect of the function of selection observed by Smeding et al. (2013) is due to the specificities of the exam situation. We argue that the function of selection is highly salient in the academic context and influences students’ every day experiences at university (as independent values which are consistently displayed while studying at university, Stephens et al., 2012a,c). As a consequence, we sought to provide initial evidence that the mere activation of the selection function is sufficient to produce the social-class achievement gap and that its effect is not restricted to exam situations *per se*.

The first goal of the present research is thus to test whether the mere activation of the function of selection without any references to the exam situation would be sufficient to produce the social-class achievement gap on a novel, non-prototypical academic task (i.e., a modular arithmetic task, based on mental calculation). A novel task was chosen in order to reduce the potential impact that students’ previous experience and performance might have on their performance in the experimental setting. While being non-prototypical in academics, modular arithmetic is based on common arithmetic operations; therefore, “it is also similar to the kinds of math problems encountered in the real world” (Beilock et al., 2004, p. 586). Further supporting the link between modular arithmetic and real-world academic tasks, good performance in this novel task relies on working memory capacity (Beilock et al., 2004)—a basic cognitive capacity used in higher-order cognitive tasks (see Engle, 2002) usually linked to achievement in several academic domains (St. Clair-Thompson and Gathercole, 2006; Packiam Alloway et al., 2010).

The second goal of the present research is to test a mechanism that might underlie the hypothesized effect of the salience of the function of selection on the performance gap between first- and continuing-generation students. Evidence from the literature shows that people who face aversive experiences are more worried about others’ performance and that this worry might explain their underperformance (Brodish and Devine, 2009; see also Smith, 2004; Chalabaev et al., 2008). Here, we argue that the underperformance of first-generation students (compared with continuing-generation students) when the function of selection is salient might be explained by a disruption of their attentional processes during the task. At the heart of this idea is the model of Schmader et al. (2008) which proposed to explain individuals’ underperformance when they are facing a stereotype threat situation through cognitive functioning impairment (see also Schmader and Johns, 2003; Croizet et al., 2004). These authors proposed different mechanisms that could impair working memory functioning and specifically a tendency to monitor one’s performance (see also Mendes and Jamieson, 2012; Schmader and Beilock, 2012). More precisely, individuals who face a threat (i.e., as hypothesized for first-generation students when the function of selection is salient) might face aversive physiological responses (e.g., cortisol secretion, Stephens et al., 2012c; blood pressure, Scheepers et al., 2009; cardiovascular responses, Murphy et al., 2007) that they will try to reduce—along with the associated uncertainty regarding their potential success—through an increased vigilance to internal and external cues informing them about their achievement.

In line with this idea, Johns et al. (2008) showed that women who faced a threatening situation focused more on anxiety-related words compared to women who did not face threat, suggesting a stronger vigilance for cues referring to their own level of anxiety (for other examples, see Williams et al., 1996; Kaiser et al., 2006; Forbes et al., 2008). Such vigilance to threatening cues (see also Davis and Whalen, 2001) could disrupt individuals’ concentration, consume cognitive resources, and impede working memory capacity (Warm et al., 2008), which can in turn reduce their performance. In the present study, individuals have the possibility to look at a visual cue that signals whether their performance is at, above, or below the mean performance level of other participants. These cues are consistently displayed during the task. The time each participant spends on each cue is measured using an eye-tracking technique. When the function of selection is salient, first-generation students should be more vigilant to cues that can confirm their inferior performance (i.e., cues signaling their performance falls below the mean level of other participants) compared to continuing-generation students—a difference that should not appear when the function of selection is not salient. This disruption of their attention should partly explain their underperformance.

In sum, in the present research, the mere activation of the selection function of the university system is examined as a minimal condition to impair first-generation students’ performance on a novel task compared to continuing-generation students’ performance. Second, a potential underlying mechanism of this effect is examined, as the salience of the selection function could enhance worries about underperformance, thereby disrupting first-generation students’ attention to the task. We hypothesized that, when the function of selection is salient (compared to when this function is not salient), first-generation students would spend more time looking at the visual cue, which should further explain their poorer performance in the task compared to continuing-generation students.

## Materials and Methods

### Participants

One hundred seventeen students enrolled in psychology at a French university voluntarily participated in the experiment in exchange for course credits. Twenty-six participants were dropped from the sample (24 for unusable eye signals and two who did not answer correctly to the experimental manipulation check). The final sample included 91 participants (70 women and 21 men) with a mean age of 18.78 years (SD = 1.30). Every participant gave his/her consent before the experiment began. An institutional ethics committee (“Comité de la Protection des Personnes Sud-Est 6”) approved the experimental protocol (Ref: 2013/CE58).

### Materials and Procedure

#### Manipulation of the Selection Function Saliency

Participants were randomly assigned to one of the two experimental conditions: a condition in which the function of selection was made salient (*N* = 43) and a condition in which its importance was reduced (*N* = 48). Indeed, as the function of selection is expected to influence students’ daily experience, it could lead participants to actually interpret a typical neutral condition—one in which no specific instructions would be provided—in terms of selection. Therefore, as in some previous studies (Smeding et al., 2013), we decided to compare the selection condition to a “no-selection” condition, where the importance of selection was explicitly reduced (for a discussion on this point, see Steele and Davies, 2003). More precisely, at the very beginning of the experiment, the study was presented as part of a state program called either “Succeeding in a bachelor program: at university, promoting excellence” (i.e., selection condition) or “Succeeding in a bachelor program: at university, success for everyone” (i.e., no-selection condition). Participants subsequently read an introductory text about the university’s functions. In the selection condition, participants read the following introduction:

“As you may know, university makes important selections. In psychology, for example, teachers do their best, throughout their practices, to identify the best students among you—those who deserve the most to become a psychologist (5 or 10% among you). In your opinion, which type of selection method should be promoted at the university in order to truly identify the best students?”

In the no-selection condition, the text was as follows:

“As you may know, university wants to give every student the opportunity to succeed. In psychology, for example, teachers do their best, throughout their practices, to help students become psychologists one day. In your opinion, which type of method should be promoted at the university to help every student succeed?”

Participants were asked to provide an answer to the final question to make sure they read the experimental inductions. Answers to this question were also used as an experimental check (cf. participants section). After answering the question, participants received a brief presentation of the task (i.e., the arithmetic modular task, see below). This task was presented as a tool either to test useful abilities to succeed in the system (i.e., in the selection condition) or to train these abilities (i.e., in the no-selection condition). Participants were reminded of the main purpose of the experiment (i.e., in the selection condition: “Your performance in this task will furnish an estimation of your probability of success at university”; in the no-selection condition: “Doing this task will allow you to train useful abilities to succeed at university”) after the training phase and immediately before the experimental phase.

#### Arithmetic Modular Task

In this experiment, participants had to complete an arithmetic modular task (Beilock and Carr, 2005; Crouzevialle and Butera, 2013; Smeding et al., 2015) in which they were asked to judge the validity of modular arithmetic problems presented as follows: “36 ≡ 12 (mod 6).” In order to solve each problem, participants had to follow two steps: (1) subtract the second number from the first and keep the result in mind (i.e., 36 – 12 = 24), and (2) divide this result by the mod (i.e., 24/6 = 4). If the result was a whole number, the problem was considered valid and the answer was “true”; if not, the problem was invalid and the answer was “false.” Participants had to answer quickly and accurately. To answer each problem, participants had to press a button on the top of one of two joysticks: the joystick in their dominant hand for the “true” responses and the joystick in their non-dominant hand for the “false” responses. As in Beilock and Carr (2005), these problems included low-demand problems (no-large operand number), intermediate-demand problems (one-large operand number), and high-demand problems (two-large operand numbers).

***Initial performance***

The first part of the arithmetic task involved a six-item training phase. The performance during this training phase (i.e., number of problems solved correctly) was used as a measure of initial performance (*M* = 4.52; SD = 1.29). It should be noted that neither the manipulation of the salience of the selection nor the generational status affected students’ initial performance (all *ps* > 0.65).

***Achievement in the task***

After the training phase, participants had to solve 48 problems presented as the main task. Their mean level of performance (i.e., number of problems correctly solved) was 37.76 (SD = 6.00). Means and standard deviations as a function of the experimental condition and generational status are presented in Table 1.

**TABLE 1:** **Means and standard deviations for achievement scores and vigilance to threat depending on the experimental condition and the generational status**.

**Condition**	**Achievement**				**Vigilance to threat**			
	**First-generation students**		**Continuing-generation students**		**First-generation students**		**Continuing-generation students**	
	*M*	SD	*M*	SD	*M*	SD	*M*	SD
Selection	35.58	6.58	39.67	5.70	4.14	3.28	3.56	2.69
No-selection	38.90	4.65	36.70	6.35	3.63	1.93	3.36	2.50

#### Generational Status

Based on previous research (Stephens et al., 2012a; Harackiewicz et al., 2014; Jury et al., 2015), parental level of education was used to assess students’ social class. Therefore, at the very end of the experiment, participants had to report their mothers’ and fathers’ highest degrees. The *baccalauréat* (i.e., the French high school exit exam) was used as the criterion for determining students’ generational status. This degree determines whether one will get access to higher education. Participants whose parents did not pass the *baccalauréat* were coded as first-generation students; if at least one parent had earned the *baccalauréat* (or any higher degree), students were classified as continuing-generation students. Based on this classification, 40 students were coded as first-generation students and 51 as continuing-generation students.

#### Participants’ Vigilance to Threat

**FIGURE 1:**
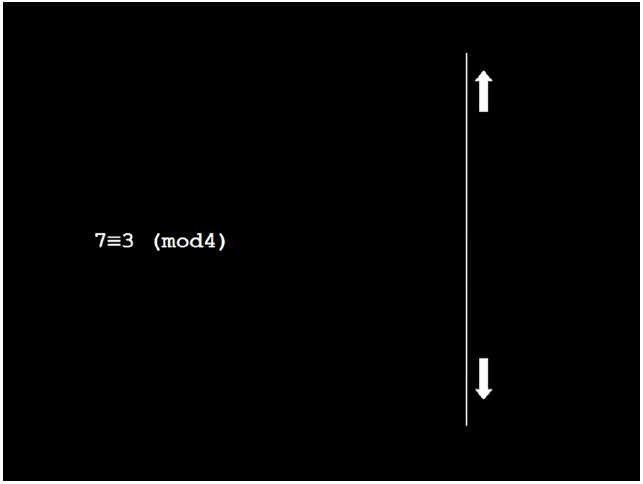
**A typical trial in the task with the MA problem on the left side and the feedback (the upward and the downward arrows) on the right side**.

In the present study, participants were allegedly informed about their performance in comparison with previous participants through two specific cues (see Figure 1). More precisely, two arrows were presented on the right part of the screen: an upward arrow oriented toward the top of the screen and a downward arrow oriented toward the bottom of the screen. Participants were told that, when the two arrows had the same size, their performance was at the mean level in comparison with other participants. When the upward arrow was bigger compared to the downward arrow, the participant’s performance was supposedly above the mean level of other participants’ performance. When the downward arrow was bigger compared to the upper arrow, the participant’s level of performance was supposedly below that of others. These cues were consistently displayed on the screen. In reality, the feedback provided was perfectly random, as our aim was to examine the extent to which participants were interested in information potentially confirming their inferiority—that is, the downward arrow.

Individuals’ vigilance was measured using an eye-tracking technique (for example, see Mogg et al., 2003). Eye movements—more precisely, eye fixations—can be considered as an indicator of individuals’ attention to various types of information (Mogg et al., 2003; Strick et al., 2009; Vainio et al., 2009; Wang, 2011; Godfroid and Uggen, 2013; Godfroid et al., 2013). In the present experiment, participants’ eye fixations were recorded using a remote eye-tracking system (i.e., iView X Hi-Speed, Senso Motoric Instruments). Movements of the two pupils were recorded continuously while participants looked at a display. Participants’ head was tuned into the eye-tracking system in order to maintain their eyes at a distance of 50 cm from the middle of the screen. Prior to the experiment, the researcher performed a calibration procedure. The data were then extracted using the SMI BeGaze software. The data from both eyes were examined independently. A judge coded each eye on a 4-point scale (from 0 = unusable to 3 = good signal). Participants for whom neither eye was coded as at least two were excluded from the sample. The signal for the best eye was kept for the analyses.

In the present experiment, three areas of interest (AOI) were defined: (1) the problem area, (2) the upward arrow area, and (3) the downward arrow area. A relative fixation time score was computed that corresponded to the fixation time in a particular AOI relative to the total fixation time in all AOIs (for examples, see d’Ydewalle and De Bruycker, 2007; Georgescu et al., 2013; Chang and Choi, 2014)

Therefore, the time participants spent looking at the three different AOIs was expressed as a percentage over the total time participants looked at the three AOIs during the experiment. The mean percentage of time spent on the problem was 88.59 (SD = 7.18), the time spent on the upward arrow was 7.78% (SD = 5.42), and the time spent on the downward arrow was 3.64% (SD = 2.59). See Table 1 for means and standard deviations as a function of the experimental condition and generational status.

## Results

ANOVAs were conducted to test the effect of the three independent variables: the experimental condition (coded –1 for selection and +1 for no-selection), the generational status (coded –1 for first-generation students and +1 for continuing-generation students), and the initial level of performance (mean-centered). The interactions among these three independent variables were also entered into the model. The initial level of performance was entered into the analyses to identify more clearly the role of our hypothesized variables (i.e., by controlling the variance explained by students’ initial abilities in the task). Preliminary analyses were conducted on the total time spent on the task (in milliseconds) as a function of the experimental condition and generational status. The results revealed that participants spent the same time on the task, regardless of the experimental condition, *F*(1,87) = 2.54, *p* = 0.12, or their generational status, *F*(1,87) = 1.91, *p* = 0.17. The interaction between these two variables was also not significant, *F*(1,87) = 2.39, *p* = 0.13. Thus, the total time spent on the task was not entered in the analyses.

### Achievement

**FIGURE 2:**
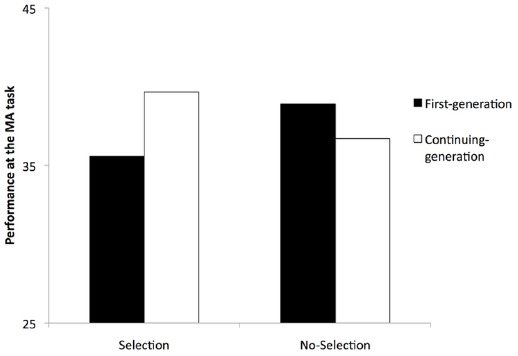
**Performance at the MA task depending on the experimental condition and the generational status**.

We hypothesized that the mere activation of the function of selection would be sufficient to impair first-generation students’ performance (number of problems correctly solved) compared to continuing-generation students. First, the results indicated that the initial level of performance predicted the level of achievement on the task, *F*(1,83) = 23.84, *p* < 0.001, ${\text{η}}_{p}^{2}$ = 0.22. Specifically, a better initial performance predicted better performance on the main task. The main effects of the experimental condition and generational status were non-significant (all *ps* > 0.51). However, the expected interaction between the experimental condition and generational status was significant, *F*(1,83) = 6.73, *p* = 0.011, ${\text{η}}_{p}^{2}$ = 0.07. As shown in Figure 2, when the function of selection was made salient, first-generation students (*M* = 35.58, SD = 6.58) performed more poorly compared to continuing-generation students (*M* = 39.67, SD = 5.70), *F*(1,83) = 4.96, *p* = 0.029, ${\text{η}}_{p}^{2}$ = 0.05. This difference did not appear when the function of selection was not salient, *F*(1,83) = 2.01, *p* = 0.16. No other interactions reached significance (all *ps* > 0.13). Although our main achievement indicator was participants’ performance on the whole set of problems, in supplementary analyses, the level of difficulty (low, intermediate, and high-demand problems) was entered in the model as a within-participants variable. The results indicated that the interaction between selection and generational status was not moderated by the level of difficulty, *F*(2,166) = 1.20, *p* = 0.30.

### Participants’ Vigilance to Threat

In the selection condition, but not in the no-selection condition, we expected first-generation students to pay more attention to the indicator that could confirm their inferiority than continuing-generation students. To test this hypothesis, we analyzed the percentage of time participants spent on the downward arrow, which indicated that they were performing more poorly relative to others. First, the main effect of initial performance indicated that the better their initial performance, the less participants looked at the downward arrow, *F*(1,83) = 12.60, *p* = 0.001, ${\text{η}}_{p}^{2}$ = 0.13. It is worth noting that a marginal interaction appeared between the experimental condition and the initial level of performance, *F*(1,83) = 3.34, *p* = 0.071, ${\text{η}}_{p}^{2}$ = 0.03. This marginal interaction suggested that the negative relationship between the initial level of performance and time spent on the downward arrow was stronger for participants in the selection condition, *F*(1,83) = 13.85, *p* < 0.001, ${\text{η}}_{p}^{2}$ = 0.14, than for those in the no-selection condition, *F*(1,83) = 1.55, *p* = 0.22. No main effect of experimental condition, of generational status, or of the interplay between experimental condition and generational status was found (all *ps* > 0.38). As an interaction between the experimental condition and generational status was not observed on the hypothetical mediator (i.e., the percentage of time spent on the downward arrow), the mediation path could not be tested.

**FIGURE 3:**
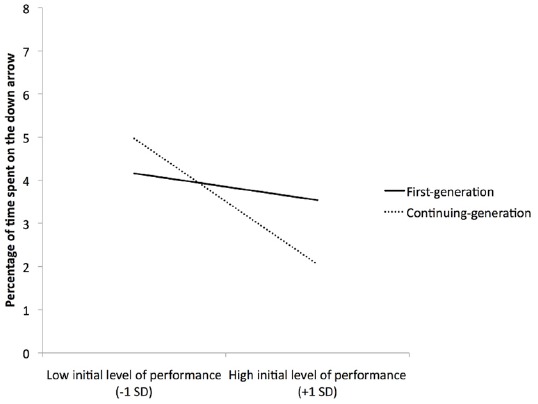
**Percentage of time spent on the downward arrow depending on participants’ generational status and the initial level of performance**.

However, an unexpected interaction between generational status and initial performance was found, *F*(1,83) = 6.07, *p* = 0.016, ${\text{η}}_{p}^{2}$ = 0.06. As shown in Figure 3, at a low level of initial performance, no difference emerged between first- and continuing-generation students *F*(1,83) = 1.73, *p* = 0.19; however, at a high level of initial performance, first-generation students were more likely than continuing-generation students to look at the downward arrow, *F*(1,83) = 4.74, *p* = 0.032, ${\text{η}}_{p}^{2}$ = 0.05. The three-way interaction did not reach significance, *p* = 0.53.

To ensure that these effects were specific to the downward arrow, analyses were also conducted on the time participants spent on the upward arrow (i.e., indicating they are performing better than others). The results revealed only a main effect of the initial level of performance—specifically, the better participants initially performed, the less they looked at the upward arrow in the second phase, *F*(1,83) = 12.91, *p* = 0.001, ${\text{η}}_{p}^{2}$ = 0.13. No other effects reached significance (all *ps* > 0.12).

## Discussion

The aim of the present research was twofold. First, the study aimed to test whether continuing-generation students outperform first-generation students on a novel academic task only when the selection function of the university system is activated. The second purpose of this experiment was to test a possible underlying mechanism that could explain this result—namely, a disruption in first-generation students’ vigilance. This mechanism is based on the hypothesis that, in a threatening situation, stigmatized people monitor their performance and are vigilant to threatening cues (Schmader et al., 2008). Thus, first-generation students were expected to spend more time on a cue informing them that they were performing more poorly compared to others when the function of selection was salient. Looking at this cue was expected to impair their performance.

As expected, first-generation students performed more poorly compared to continuing-generation students when the function of selection was salient. In the no-selection condition, no differences were found. These results replicated the findings obtained in previous work conducted in an academic exam situation on a typical academic task (Smeding et al., 2013), tending to confirm that the university system can create a threatening climate (i.e., through its function of selection) that leads first-generation students to be outperformed by continuing-generation students. It is important to note that the present results extend previous knowledge by showing that such differences can be observed in minimal conditions, via the mere activation of the function of selection, and are not restricted to typical exam situations. The findings also provide support for the idea that, despite an institutional discourse claiming equal opportunities for every student, the very functioning of the university system contributes to continuing-generation students’ better performance.

Regarding the second purpose of this work, we expected that, in a threatening situation, stigmatized individuals should monitor their performance and be more vigilant to threatening cues—namely, to cues that might confirm their inferiority. To test these hypotheses, we measured students’ vigilance for a threatening cue—that is, an arrow indicating that they were underperforming compared to others—via their eye movements. The results did not support the hypothesis. Indeed, no difference appeared between first- and continuing-generation students’ attention to the downward arrow, regardless of the experimental condition. This lack of interaction on the hypothesized mediator did not allow to further test the potential mediation path assuming that the vigilance disruption may explain first-generation students’ underperformance.

Different explanations might contribute to our understanding of why, in this experiment, no evidence was found to support the hypothesis that first-generation students were more vigilant to the threatening cue (i.e., the downward arrow) compared to continuing-generation students when the function of selection was salient. First, Van Yperen and Leander (2014) demonstrated that, because social comparison information is prevalent in our society (i.e., excellence and success are often defined in comparison with others; see Harackiewicz et al., 1998), comparison with others is an automatic process (see also Gilbert et al., 1995). Consequently, students in the present experiment might have looked at the social comparison information on the screen even in the no-selection condition because this information is readily available and difficult to avoid (i.e., as it results from an automatic process). Another explanation might be that students do not usually receive direct (i.e., online) feedback when they are working on an academic task. Thus, providing real-time feedback might appear as a relatively novel type of information and act as a disruptor, encouraging every student (i.e., regardless of experimental condition and generational status) to pay attention to the available normative information. Moreover, although often used in previous research, the eye-tracking measure used in the present research might not be precise enough. Indeed, by estimating the total time spent on three different AOIs, we obtained a global score of behavior that might not identify students’ sheer intentions or interests by including some artificial “noise” (i.e., students who looked at the arrows without thinking about their performance, instead thinking about how to solve the problem). One promising perspective to go beyond this possible limitation would be to design a study that would allow us to identify students’ first intention for each problem. By isolating students’ eye movements in the first milliseconds of each trial, as done by Beattie and McGuire (2012), it could be possible to identify whether students are first interested in the problem and/or in the threatening cue.

However, the results concerning the vigilance to the threatening cue provided interesting information. Indeed, the results showed that first-generation students with a high level of initial performance were more vigilant to the downward arrow than their continuing-generation counterparts. In other words, first-generation students whose initial level of performance was high seemed more concerned about performing poorly than their continuing-generation counterparts. These results can be linked to recent research in the achievement goal literature (Jury et al., 2015). Indeed, the achievement goal literature assumes that, when facing an academic task, students can pursue different types of achievement goals (Elliot et al., 2011), including performance-avoidance goals (i.e., defined as the goal not to perform poorly in comparison with others). Recently, it has been argued that first-generation students with a high level of academic achievement are particularly prone to endorse performance-avoidance goals in comparison with continuing generation students because they are close to achieve an upward mobility process, a process that can be costly (Jetten et al., 2008; Reay et al., 2009; Lee and Kramer, 2013). Indeed, these authors proposed that the identity-threat that first-generation students might face at university should be even more salient for competent first-generation students because of the conflict between their actual identity (i.e., first-generation students) and their prospective identity (i.e., a higher-status one). Results from three studies confirmed that, at a high level of academic achievement, first-generation students endorsed more performance-avoidance goals than their continuing-generation counterparts. In a different yet complementary way, the results obtained in the present experiment tend to confirm these findings by showing that first-generation students, despite a high initial level of performance, pay more attention to the downward arrow, suggesting they are more concerned about performing poorly compared to continuing-generation students. Such motives (i.e., the fear of performing more poorly than others) have been shown to be highly detrimental for students (e.g., low intrinsic motivation, Elliot and Church, 1997; disorganization, surface learning, Elliot and McGregor, 2001; poor academic performance, Van Yperen et al., 2014); thus, the present results open questions on how first-generation students deal with a high level of performance. Indeed, a high level of academic performance should be a force that orients individuals toward positive perspectives (i.e., like approach forms of motivation, see Cury et al., 2006). The present results suggest that it can rather be an additional burden for first-generation students. An explanation of this process could be that competent first-generation students are not able to correctly estimate their level of competence and might perceive it as relatively low (for an example, see Ivcevic and Kaufman, 2013). This potential low level of perceived competence could explain why these students are more oriented toward avoidance forms of motivation (Cury et al., 2006). Future research should address this question in order to understand this process more clearly and to propose interventions that could help these students stay away from avoidance tendencies.

Despite its contributions, the results of the present experiment should be interpreted with caution for several reasons. First, in the present experiment, the mere activation of the function of selection condition was not compared with a neutral control condition, but with a condition in which the importance of the function of selection was reduced. In particular, the no-selection condition focused on improvement and training, a type of instruction that might be beneficial to low-status people (Aronson et al., 2002; Souchal et al., 2014). Consequently, it is difficult to know whether the effect is due to the increase of the selection function saliency, the decrease of this saliency in the no-selection condition, or both. Second, in the present research, the selection condition reported a very high selection rate (i.e., 5 to 10%). If anything, this rate might have strengthened the effects by making the social identity threat more salient (i.e., the higher the selection is, the tougher success is and the lower low social-class students’ feeling of legitimacy might be). If such a rate corresponds to the selection practices in several universities, it could vary a lot across countries, fields, and types of universities. In order to generalize our findings, the rate of selection should be varied experimentally in future research. Finally, the present findings apply to the arithmetic modular task examined here. Replicating the findings on different academic tasks would strengthen their generalizability.

Notwithstanding these limitations and the need to carefully establish conclusions, we believe that the present research offers a substantial contribution to the literature for several reasons. First, by showing that when the function of selection was salient first-generation students underperformed compared to their continuing-generation counterparts, this experiment tends to confirm that—despite an institutional discourse claiming equality between students, regardless of their social class background—the university system might contribute to the social reproduction phenomenon (Bourdieu et al., 1990; Stephens et al., 2012a; Smeding et al., 2013) and the mere activation of this function seems to be sufficient to threaten first-generation students. Second, although more data are needed to confirm this effect, part of the results seems to confirm that, at a high level of performance, first-generation students are more worried about failure than continuing-generation students. These results support the general hypothesis that coming from a low social-class background is challenging at university (Stephens et al., 2015). Previous work showed that lower low social-class students face a lot of negative outcomes when attending university (e.g., a lower sense of belonging, Ostrove and Long, 2007; a lower level of self-efficacy, Ramos-Sánchez and Nichols, 2007; a higher feeling of guilt, Covarrubias and Fryberg, 2014; a higher level of depression, Stebleton et al., 2014). The present work extended previous research (Jury et al., 2015) by showing, within a different paradigm, that these difficulties seem to be particularly experienced by high achievers.

From an applied perspective, these latter results could be another argument sustaining researchers’ rising interest in interventions aimed at improving first-generation students’ experience at college. Indeed, as previously developed, accumulated evidence from the literature emphasizes how difficult the path to graduation can be for these students, leading Stephens et al. (2015) to propose different kinds of interventions that might help first-generation students have better experiences/more success at college. The present results, showing that the achievement gap between first- and continuing-generation students can be eliminated when the importance of the function of selection is reduced, could bolster this need for interventions. Previous work has shown that student-based interventions, such as a self-affirmation technique (Harackiewicz et al., 2014) or a difference-education intervention (Stephens et al., 2014), could significantly reduce the magnitude of the achievement gap. The present research suggests that acting directly on the meaning of the situation (e.g., promoting at an institutional level the idea that university aims to provide each student with the opportunity to succeed) can also significantly reduce the achievement gap.

## Author Contributions

MJ, AS, and CD conceived and designed the study. MJ collected the data and analyzed it under the supervision of AS and CD. MJ drafted the manuscript and AS and CD provided critical revisions. All authors approved the final version of the manuscript for submission.

### Conflict of Interest Statement

The authors declare that the research was conducted in the absence of any commercial or financial relationships that could be construed as a potential conflict of interest.
